# Multiple GPCR conformations and signalling pathways: implications for antagonist affinity estimates

**DOI:** 10.1016/j.tips.2007.06.011

**Published:** 2007-08

**Authors:** Jillian G. Baker, Stephen J. Hill

**Affiliations:** Institute of Cell Signalling, Medical School, Nottingham, NG7 2UH, UK

## Abstract

Antagonist affinity measurements have traditionally been considered important in characterizing the cell-surface receptors present in a particular cell or tissue. A central assumption has been that antagonist affinity is constant for a given receptor–antagonist interaction, regardless of the agonist used to stimulate that receptor or the downstream response that is measured. As a consequence, changes in antagonist affinity values have been taken as initial evidence for the presence of novel receptor subtypes. Emerging evidence suggests, however, that receptors can possess multiple binding sites and the same receptor can show different antagonist affinity measurements under distinct experimental conditions. Here, we discuss several mechanisms by which antagonists have different affinities for the same receptor as a consequence of allosterism, coupling to different G proteins, multiple (but non-interacting) receptor sites, and signal-pathway-dependent pharmacology (where the pharmacology observed varies depending on the signalling pathway measured).

## Introduction

The measurement of antagonist affinity has traditionally been an important feature of the characterization of cell surface receptors [1–5] and has been used to identify novel receptor subtypes [1,3,6,7]. A central assumption of this approach is that antagonist affinity is constant for a given receptor–antagonist interaction, regardless of the agonist used to stimulate that receptor or the downstream response that is measured [6]. However, there is emerging evidence that receptors can have multiple binding sites and that the same receptor can exhibit different antagonist affinity measurements under different experimental conditions [8–13]. It might, therefore, be timely to ‘rethink’ this basic concept in pharmacology.

## Classical analysis of functional agonist–antagonist interactions

**Figure 1:**
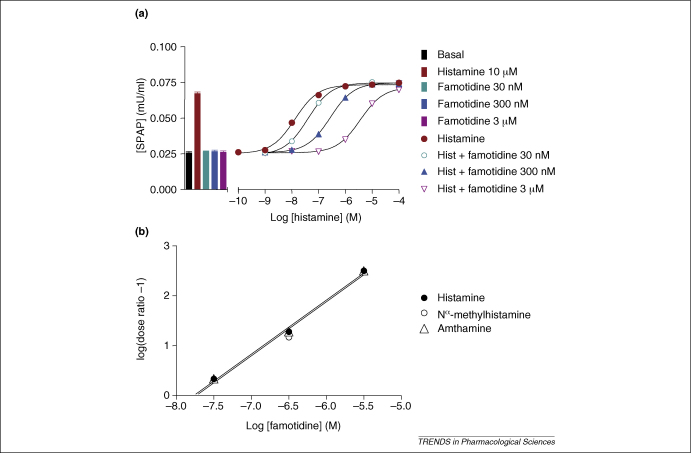
Antagonism of histamine H2 receptor responses. **(a)** Antagonism of histamine-stimulated CRE (cAMP response element) gene transcription, mediated through the H_2_ receptor, by increasing concentrations of the H_2_ antagonist famotidine in CHO cells expressing the human H_2_ receptor. Gene transcription was measured by using a secreted placental alkaline phosphatase (SPAP) reporter gene. **(b)** Schild plots of the famotidine antagonism of H_2_ responses stimulated by histamine, *N*^α^-methylhistamine and amthamine. The *x*-axis intercept gives the −log *K*_b_ value. Data are from Ref. [16] and J.G.B. (unpublished observations).

Analysis of logarithmic concentration–response curves has long been used to evaluate the nature of the competitive interactions between agonists and antagonists from functional measurements and particularly so in the case of G-protein-coupled receptors (GPCRs) (Figure 1). The standard approach is the construction of full agonist concentration–response curves in the absence and presence of fixed concentrations of antagonist. The extent of the parallel rightward shift in the position of the agonist concentration–response curve is then used to calculate the antagonist affinity directly (assuming competitive antagonism) or (if various antagonist concentrations have been used) to construct a Schild plot, which will have a slope of one if the interaction is competitive (Figure 1).

The principal assumptions made in these calculations are (i) that there is only one binding site; (ii) that the agonist and the antagonist are competing at this same site; and (iii) that, for a given level of response, the agonist occupancy of the receptors will be identical in the presence and absence of antagonist [1]. This latter assumption is required to take into account the two fundamental properties of an agonist: namely, affinity and efficacy, which contribute to the final measured response [14,15]. Provided that the agonist–antagonist interaction with the receptor is competitive, then the ratio of agonist concentrations required to give the same-sized functional response in the presence and absence of antagonist is equal to 1 + *BK*_b_, where *B* is the concentration of antagonist and *K*_b_ is the antagonist affinity constant [1] (Figure 1). For a given receptor, the Kb value derived from this relationship should be independent of the agonist used to stimulate the receptor and the level in the receptor signalling cascade at which responses are measured. For example, in the case of the histamine H_2_ receptor, affinity constants for the H_2_ antagonist famotidine are the same whether histamine, amthamine or N^α^-methylhistamine are used as H_2_ receptor agonists [16] (Figure 1).

## Allosteric antagonism

It has been accepted for some time that some GPCRs (most notably muscarinic, adenosine and CCR5 receptors) have more than one binding site [17–21]. Endogenous ligands bind to and activate the orthosteric site, whereas others, known as ‘allosteric ligands’, bind to a separate site or sites on the same receptor. As a consequence, both the orthosteric and allosteric sites can be occupied by ligands at the same time.

Ligands binding to the allosteric site can alter the binding of ligands to the orthosteric site and have traditionally been considered not to induce a receptor response on their own. Thus, a positive allosteric regulator binds to the allosteric site and increases the binding affinity of an orthosteric ligand, causing the concentration–response curve of an orthosteric agonist to move leftward to lower agonist concentrations. A negative allosteric regulator decreases the binding of the orthosteric ligand such that either the orthosteric agonist concentration–response curve will be shifted to higher agonist concentrations (i.e. rightward) or the maximum response will be decreased (depending on the signalling efficiency of the orthosteric site). In this situation, the allosteric ligand is effectively acting as an allosteric antagonist.

The nature of the antagonism produced by an allosteric antagonist is therefore very different from the classic competitive antagonism described earlier (where the agonist and antagonist compete for binding to the same orthosteric site), which has several important consequences. When all of the allosteric sites are occupied, the orthosteric agonist concentration–response curve cannot be shifted any further to the right and will reach a limiting value. This can lead to nonlinear Schild plots and to incomplete displacement of radioligands from their specific binding sites [10].

The effect of the allosteric antagonist can vary markedly depending on the agonist under study [10]. For example, the M2 muscarinic allosteric ligand eburnamonine enhances the agonist affinity of pilocarpine at the orthosteric site, has no net effect on the binding affinity of arecaidine propargyl ester, but reduces the agonist affinity of arecoline [10,22]. In addition, the magnitude of the shift of agonist affinities varies not only between agonists but also with different allosteric ligands, even at the same allosteric site [10]. Thus, each ‘allosterically occupied’ form of the receptor can be considered to represent a conformationally altered receptor that will have its own unique set of ligand affinities and efficacies [10,21,22].

## Different G-protein-coupled states of GPCRs

Increasing evidence indicates that GPCRs can couple to more than one G protein, raising the possibility that different agonists can direct signalling from the receptor to specific signalling cascades as a consequence of their relative affinities for different G-protein-coupled states of the same receptor [13,23,24]. This possibility was first realized for the 5-HT_2C_ receptor, which in Chinese hamster ovary (CHO) cells can couple to two different signalling pathways (phosphoinositide hydrolysis and arachidonic acid release) with a different rank order of agonist efficacies [23]. It is equally possible, however, that antagonists might differ in their affinity for these G-protein-specific states of the receptor, which will be manifest in both agonist and signalling-pathway-dependent pharmacology.

**Figure 2:**
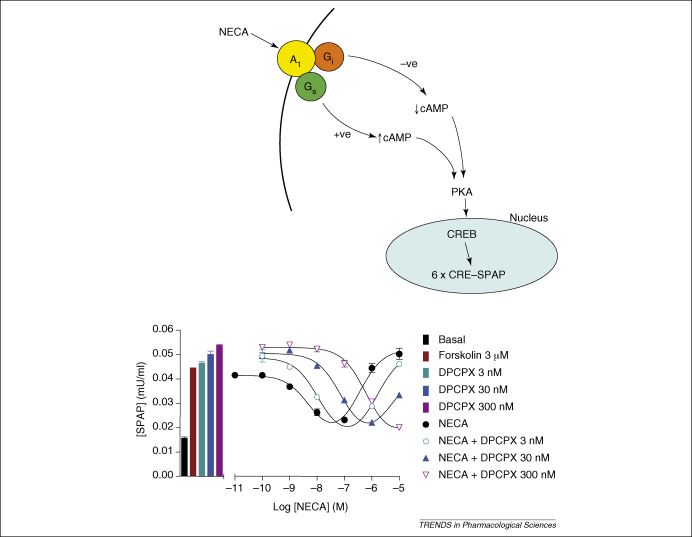
5′-N-ethylcarboxamidoadenosine (NECA)-induced gene transcription mediated by the human A1 adenosine receptor. **(a)** G_i_ and G_s_ signalling pathways from the adenosine A_1_ receptor to CRE-mediated gene transcription in CHO cells expressing the human A1-receptor. Abbreviations: CREB, CRE-binding protein; PKA, protein kinase A. 6 × CRE–SPAP signifies a SPAP reporter gene containing six CRE elements. **(b)** Concentration–response curves for the effect of the agonist NECA on forskolin-stimulated CRE gene transcription in the presence and absence of increasing concentrations of DPCPX. Data are from Ref. [26].

When expressed in CHO cells, the human adenosine A_1_ receptor couples to both G_i_ and G_s_ proteins [24,25]. When antagonist affinity measurements were made at both the G_i_- and G_s_-coupled forms of the A_1_ receptor, however, the antagonist affinities were found to be constant, regardless of the signalling cascade that was monitored (G_i_ or G_s_) or the level at which the signalling cascade was evaluated (cAMP or CRE-mediated gene expression) [26] (Figure 2). Thus, in this study the fundamental pharmacological concept that antagonist affinities are indeed constant seems to hold true for the A_1_ receptor.

This concept might not, however, apply to all GPCRs and GPCR–G-protein complexes; indeed, studies of large libraries of ligands and multiple signalling pathways might be required to confirm or to refute it. Likewise, antagonist affinity can vary for the monomeric GPCR as compared with a dimeric or multimeric complex. Similarly, if a complex is held in a scaffold (whether or not this scaffold signals to different G proteins), then the antagonist affinity might vary depending on the make-up of the specific complex or scaffold in each cell type.

Because it is becoming increasing clear that specific GPCR–G-protein signalling complexes seem to be compartmentalized in microdomains within the same cell [27,28], single-molecule approaches might be required to determine antagonist affinities in specific microdomains of single living cells [29,30].

## Non-interacting conformations of the same GPCR

The large number of ligands available for the β-adrenoceptor family of GPCRs has enabled detailed studies to be performed on these receptors. For example, there is now strong evidence that the human β_1_ adrenoceptor has at least two ligand-binding sites, each with unique pharmacological properties [31–33]. These sites are distinguishable by the ability of β-antagonists to show markedly different antagonist affinities dependent on the agonist being used to stimulate the β_1_ adrenoceptor [31].

Initial evidence for multiple binding sites on the β_1_ adrenoceptor came from detailed studies with CGP 12177. This compound is a high-affinity neutral antagonist of the classical ‘catecholamine’-binding site of the β_1_ adrenoceptor; however, at higher concentrations it produces an agonist response that is relatively resistant to antagonism by other classical β-antagonists [34–36]. The unique pharmacological properties of the agonist actions of CGP 12177 in producing cardiostimulant effects in the heart first led to the suggestion that a novel β_4_ adrenoceptor was responsible for these effects [37]. However, the loss of this ‘β_4_’ activity in β_1_ adrenoceptor knockout mice, coupled with the demonstration of ‘β_4_’ pharmacology in cells transfected with only the β_1_ adrenoceptor, led to the acceptance that the β_1_ adrenoceptor has an obligatory role in the expression of this unexpected pharmacological profile [35,38].

Several studies have now examined in detail the ability of different ligands to interact with the high-affinity ‘catecholamine site’ and the low-affinity ‘CGP 12177 site’ [8,11,35,36]. Classical catecholamines such as isoprenaline and adrenaline act as agonists of the catecholamine site, whereas CGP 12177, LY 362884 and carvedilol have agonist actions at the secondary CGP 12177 site [8,11,35,36,39]. In addition, some compounds (e.g. alprenolol and pindolol) have agonist actions at both sites [8].

**Figure 3:**
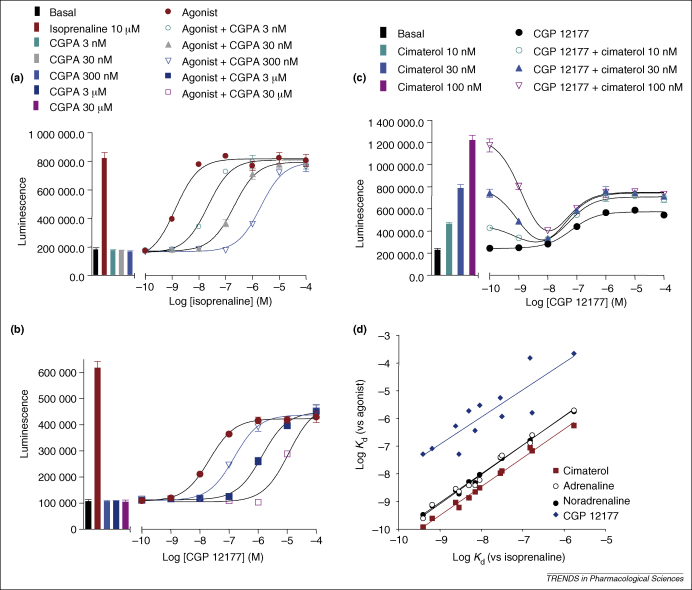
Differential affinities of antagonists for the ‘catecholamine’ and ‘CGP 12177’ sites of the human β_1_ adrenoceptor expressed in CHO cells. **(a,b)** Effect of increasing concentrations of the selective β_1_ adrenoceptor antagonist CGP 20712A (CGPA) on isoprenaline-stimulated (a) and CGP-12177-stimulated (b) CRE-mediated luciferase expression. Note that the blue data points and line in (a) and (b) represent the same concentration of CGP 20712A (300 nM). **(c)** CRE–luciferase response to CGP 12177 in the absence and presence of fixed concentrations of cimaterol. **(d)** Correlation between the logarithm of the antagonist dissociation constant (log *K*_d_) obtained for 12 antagonists with the agonist isoprenaline (*x* axis) and log *K*_d_ determined with the same 12 antagonists but with adrenaline, noradrenaline, cimaterol or CGP 12177 as the agonist (*y* axis). Data are from Refs [8,11].

The agonist dependence of the antagonist affinity estimates is perhaps best illustrated by a correlation plot of the values obtained for a range of different antagonist affinities when several different ligands were used as agonists [8,11] (Figure 3). Although all antagonists have low affinity for the CGP 12177 site, the rank order of affinities varies between the two sites; for example, some compounds (e.g. atenolol) differ by up to 1000-fold in their affinities for the two sites, whereas others (e.g. ICI 118551) differ by only a factor of 10 [11]. Schild analysis of the ability of the β_1_ adrenoceptor antagonist CGP 20712A to antagonise the responses at both sites indicated that both interactions are perfectly described by a competitive interaction yielding a Schild slope of 1 [8] (Figure 3a,b). This finding strongly suggests that the two sites are completely separate and non-interacting. Furthermore, because CGP 12177 is a high-affinity antagonist of the catecholamine site (site 1), binding of CGP 12177 to the two sites of the receptor can be observed in a single experiment.

Figure 3c shows that low concentrations of CGP 12177 inhibit cimaterol (a site-1 agonist), whereas higher concentrations of CGP 12177 stimulate an agonist response (through site 2). These effects cannot simply be explained by an allosteric regulation by CGP 12177 of the orthosteric binding site [8,11,34,35]. Also plotted in Figure 3 is the correlation of the antagonist affinity (log *K*_d_) of 12 antagonists, determined in the presence of different agonists (Figure 3d). The log *K*_d_ value of the antagonists is the same whether isoprenaline, adrenaline or noradrenaline is used as the agonist (thus, the points are indistinguishable). The *K*_d_ values obtained for the same 12 antagonists when CGP 12177 is used as the agonist are clearly very different, and the rank order (seen as the pattern of scatter) does not parallel that of the catecholamines. Lastly, although the antagonist affinities measured when cimaterol is the agonist are in the same rank order, the values obtained are consistently higher than those obtained when the catecholamines are present. This observation raises the possibility that there might be more than two ‘sites’ on the β_1_ adrenoceptor, or at least more than one conformation of the catecholamine site.

The data obtained with the human β_1_ adrenoceptor firmly establish the concept that there might be additional ligand-binding sites on GPCRs that are separate from the classical orthosteric site and that can stimulate functional responses. These sites do not necessarily need to interact cooperatively and they might provide an alternative means by which cell signalling can be initiated with a completely different pharmacological profile and possibly by a completely different set of ligands to those of the orthosteric site. Furthermore, the data obtained with the β_1_ adrenoceptor also beg the question of how widespread this phenomenon is. Possibly, we have not found other agonist sites on other GPCRs because we have not looked for them or because our high-throughput screening strategies are too focused on the traditional view of the orthosteric site.

A detailed study of the human β3 adrenoceptor set out to test precisely this possibility. Early suggestions for multiple states of the human β3 adrenoceptor had been put forward to explain the apparently opposite relative potencies of β3 agonists obtained when evaluated by cAMP measurements in intact cells and when measured by radioligand binding studies in membrane preparations [40]. The differences in antagonist affinity observed at the β3 adrenoceptor were not as pronounced as those at the β_1_ adrenoceptor; however, some agonists were clearly inhibited more potently by antagonists than were other ligands [41]. Furthermore, ZD 7114 was shown to be able to activate two conformations or sites of the β3 adrenoceptor (analogous to alprenolol and pindolol at the β_1_ adrenoceptor) and alprenolol was found to be a neutral antagonist of one site while activating a second site (similar to CGP 12177 at the β_1_ adrenoceptor) [41].

## Signalling-pathway-dependent pharmacology

As their name suggests, the main signalling pathways by which GPCRs have normally been thought to function is by activation of heterotrimeric G proteins. Recent studies, however, have provided evidence for G-protein-independent signalling by the β_2_ adrenoceptor, vasopressin V_2_, parathyroid hormone and angiotensin AT_1_ receptors [9,42–48]. The potential for GPCRs to form complexes with signalling proteins other than G proteins also raises the possibility that agonists and antagonists can discriminate between these complexes in terms of both binding affinity and efficacy. Thus, each downstream signalling pathway measured in a particular cell might have its own unique pharmacology depending on the pathway stimulated by each unique ligand–receptor conformation or complex involved.

**Figure 4:**
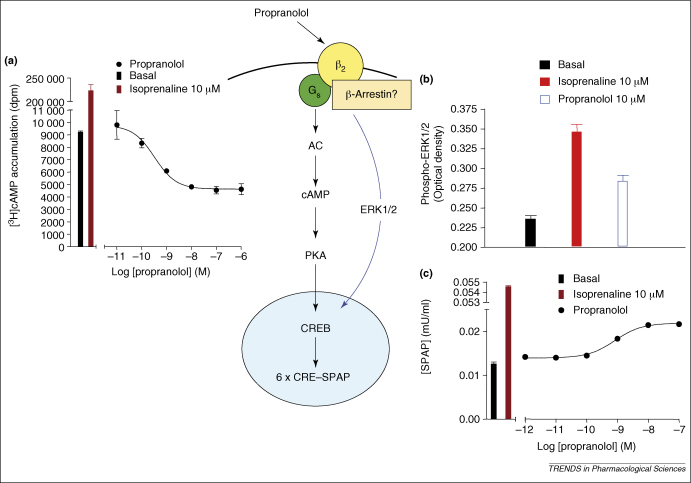
Dual efficacy of propranolol on β2-adrenoceptor-mediated responses in CHO cells expressing the human β_2_ adrenoceptor. **(a)** Inverse agonist effect of propranolol on [^3^H]cAMP accumulation. 6 × CRE–SPAP signifies a SPAP reporter gene containing six CRE elements. **(b)** Agonist effects of isoprenaline and propranolol on levels of phosphorylated ERK1/2 monitored by an ELISA assay. **(c)** Agonist effect of propranolol on CRE-mediated gene transcription. Data are from Ref. [9].

Some insight into the potential for the human β_2_ adrenoceptor to exist in different conformations (each with its own unique pharmacology) and to couple to distinct signalling pathways has been provided by detailed studies of the agonist and inverse agonist properties of propranolol on activation of the ERK1/2 mitogen-activated protein (MAP) kinase pathway and cAMP accumulation in CHO and human embryonic kidney 293 (HEK293) cells [9,43,49] (Figure 4). In the case of cAMP accumulation in CHO or HEK293 cells expressing the human β_2_ adrenoceptor, propranolol shows clear inverse agonist properties [9,43,49]. When ERK1/2 activation is measured in the same cells, however, propranolol behaves as a partial agonist. It also seems that stimulation of ERK1/2 phosphorylation by propranolol is independent of G_i_ or G_s_ protein activation [9,43]. Furthermore, it has been shown in HEK293 cells that ERK1/2 stimulation involves an interaction with β-arrestins [43]. These data suggest that ligand-specific conformations of the β_2_ adrenoceptor do indeed exist that can differentially activate distinct signalling pathways with very different pharmacologies. A similar observation has also been made for the murine β3 adrenoceptor, for which SR59230A is an antagonist of CL316243-mediated increases in cAMP accumulation in adipocytes, but is an agonist with greater efficacy than CL316243 for extracellular acidification in the same cells [50].

It is therefore possible that antagonists might differ in their affinity for different GPCR–signalling protein complexes (e.g. G_s_- and β-arrestin-coupled forms of the β_2_ adrenoceptor) and show agonist- and signalling-pathway-dependent affinities. In this respect, it is interesting that the affinity constants for different β_2_ adrenoceptor antagonists obtained in gene transcription studies in cells expressing the β_2_ adrenoceptor were found to differ by a factor of 10 depending on the competing agonist [51]. The exact mechanisms underlying this observation, however, remain to be established.

## Other receptors with different ‘pharmacological’ antagonist affinities

Comparison of the antagonist affinities estimated from radioligand binding studies and those estimated from functional studies with the same ligands shows that there are major discrepancies in the affinities obtained at the β_1_ adrenoceptor. For example, binding of ^3^H-labelled CGP 12177 gives a receptor affinity for propranolol of 6.9 nM; when propranolol antagonises the CGP 12177 agonist response in functional assays, however, the affinity obtained is 363 nM [11,52]. This discrepancy arises because the low concentration of ^3^H-labelled CGP 12177 used in the binding assay is measuring site-1 binding (there is not sufficient ^3^H-labelled CGP 12177 present to occupy site 2), whereas the CGP 12177 agonist response is occurring at site 2 and therefore propranolol affinity at site 2 is being measured. Antagonist affinities measured in functional assays with site-1 agonists correlate well with the binding studies. Comparing similar data from other receptors is a means by which insight might be gained into how widespread the presence of multiple binding sites might be in GPCRs.

For example, the α_1L_ adrenoceptor might represent an alternative conformation of the α_1A_ adrenoceptor [53–55]. The α_1L_ adrenoceptor in functional studies in native tissues has a unique pharmacological profile that is characterized by lower affinities for prazosin, WB 4101, 5-methylurapidil and *S*-niguldipine than would be predicted from studies of ligand binding to the α_1A_ adrenoceptor [53,56,57]. Other ligands such as tamsulosin and indoramin, however, yield identical binding affinities between the two assays. Expression of the cloned human α_1A_ adrenoceptor in CHO cells, and comparison of antagonist affinities between ligand binding studies and functional noradrenaline-stimulated inositol phosphate responses, has yielded α_1A_ adrenoceptor (binding) and α_1L_ adrenoceptor (functional) pharmacologies [53,54]. It is possible, therefore, that the presence of multiple binding sites on the α_1A_ adrenoceptor (analogous to those on the human β1 and β3 adrenoceptor) or signalling-pathway-dependent α_1A_ adrenoceptor conformations might explain these discrepancies.

## Concluding remarks

It is now clear that, for many GPCRs, antagonist affinities can no longer be assumed to be always constant for a particular receptor. Antagonist affinities can vary depending on the agonist that they are counteracting, the presence or absence of allosteric ligands, the specific site on the GPCR through which they exert their effect, and the specific signalling pathway under consideration. The fact that antagonist drugs can have differential effects at different sites on the same receptor means that we should no longer simply think in terms of ‘class effects’ of receptor antagonists. This concept is particularly important when we consider ‘antagonist’ drugs that can manifest different effects on specific signalling cascades through a single receptor within the same cell.

Instead, we should explore the huge potential provided by multiple binding sites and divergent signalling cascades from the same GPCR, while at the same time exploiting quantitative analytical approaches and novel screening designs to re-evaluate how widespread these phenomena are.
